# The Effects of Dietary Interventions on Brain Aging and Neurological Diseases

**DOI:** 10.3390/nu14235086

**Published:** 2022-11-30

**Authors:** Fleur Lobo, Jonathan Haase, Sebastian Brandhorst

**Affiliations:** Longevity Institute, School of Gerontology, Dept. of Biological Sciences, University of Southern California, 3715 McClintock Avenue, Los Angeles, CA 90089-0191, USA

**Keywords:** intermittent fasting, periodic fasting, brain, aging, neurological diseases, Alzheimer’s, Parkinson’s, epilepsy, multiple sclerosis

## Abstract

Dietary interventions can ameliorate age-related neurological decline. Decades of research of in vitro studies, animal models, and clinical trials support their ability and efficacy to improve behavioral outcomes by inducing biochemical and physiological changes that lead to a more resilient brain. Dietary interventions including calorie restriction, alternate day fasting, time restricted feeding, and fasting mimicking diets not only improve normal brain aging but also slow down, or even reverse, the progression of neurological diseases. In this review, we focus on the effects of intermittent and periodic fasting on improving phenotypic outcomes, such as cognitive and motor-coordination decline, in the normal aging brain through an increase in neurogenesis and synaptic plasticity, and decrease in neuroinflammation, mitochondrial dysfunction, and oxidative stress. We summarize the results of various dietary interventions in animal models of age-related neurological diseases such as Alzheimer’s disease, Parkinson’s disease, epilepsy, and Multiple Sclerosis and discuss the results of clinical trials that explore the feasibility of dietary interventions in the prevention and treatment of these diseases.

## 1. Introduction

The aging related onset of cognitive disabilities and neurological diseases such as Alzheimer’s (AD), Parkinson’s (PD), Epilepsy, and Multiple Sclerosis (MS) is a complex multifactorial process that results in subsequent physiological, psychological, and behavioral changes. According to the Center for Disease Control *“Dementia is not a specific disease but is rather a general term for the impaired ability to remember, think, or make decisions that interferes with doing everyday activities.”* AD is the most common cause of dementia, present in 60–80% of cases, followed by Lewy Body Disease (LBD), frontotemporal and vascular dementia, and others [1,2]. Although PD is not a form of dementia, it is a neurodegenerative disease, and 75% of those who survive for over 10 years with the condition will eventually develop dementia [3]. Mixed dementia in which neuropathologies overlap is common, and different diseases may also have related characteristics. For instance, AD pathology is present in 10–59% of patients with LBD, with synergizing effects that may act on distinct cognitive domains [4]. Another example is found in PD and LBD, distinct disorders that both involve the aggregation of lewy bodies in the brain made up of α-synuclein [5].

Neurological diseases may appear early in life and do not always result in dementia but may still be associated with an increased risk for age-related cognitive decline. As such, epilepsy includes different pathologies that share the common characteristics of unprovoked and recurrent seizures. Epilepsy commonly develops either in infancy or over the age of 60 [6]. Age-related epilepsy can be caused by a wide variety of etiologies, and at least 40% of adult-onset cases of epilepsy have no known cause [7,8]. AD is 6 times more common in people with epilepsy than in the general population [9], and vice versa dementia and neurodegenerative disorders may be the driving factor behind 10–20% of epilepsy diagnoses [10].

Multiple sclerosis (MS) is a neuroinflammatory disease that often leads to progressive neurodegeneration. Recovering Remitting Multiple Sclerosis (RRMS), where periods of inflammatory attacks are followed by remission and recovery, is the most common of the 4 main types of MS. RRMS is usually diagnosed between the ages of 20–50 and is prevalent in 87% of patients [11,12]. Nearly 65% of RRMS patients will eventually progress to the Secondary Progressive phase (SPMS), which is characterized by the gradual and irreversible loss of physical and cognitive abilities [13]. The cognitive impairment and decline that accompanies the later stages of SPMS is not classified as dementia, because it is generally not as severe and follows a different pattern than the impairment found in AD [14,15]. Although distinct from dementia, MS is also an age-related neurodegenerative disease, and people over the age of 65 are more likely to have developed SPMS [16].

Modifying lifestyle factors may prevent or delay up to 40% of dementia cases, and a recent 2020 Lancet commission on dementia prevention and intervention identified 12 risk factors, including hypertension, obesity, and diabetes [17]. Similarly, childhood obesity has been shown to be a risk factor for MS in studies across different countries [18,19,20].

Dietary factors such as the number of calories consumed, the macronutrient composition, or when meals are consumed present modifiable risk factors that are commonly altered to investigate the effects on healthy aging and the prevention of age-related diseases [21]. Furthermore, these factors may also present tools that can be used as therapeutic interventions not only to prevent the development of aging-related diseases, but also to treat symptoms after diseases are established [22]. Different methods of caloric restriction (CR) and dietary restriction (DR) have been shown to modulate evolutionary conserved and interrelated nutrient-sensing mechanisms of IGF-1, AMPK, and mTOR in cells, thereby extending lifespan and prevent aging-related diseases in animals and humans [23].

CR generally refers to the reduction of calories without malnutrition whereas DR refers to the reduction of specific dietary components without malnutrition, for example protein restriction (PR) or ketogenic diets (KD) which are high in fat but with minimal carbohydrates to induce long-term ketosis. Although they are not the focus of this paper, KDs are included in many of the reviewed studies because they have some overlapping effects with CR and fasting, and often are compared against them [24].

Fasting studies utilize varying periods of time where food intake is completely stopped or severely limited. Intermittent Fasting (IF) describes different patterns of fasting for 24 h or less, and includes alternate day fasting (ADF), 5:2 IF, and time restricted eating/feeding (TRE, or TRF for mice). ADF is complete or partial fasting every other day, 5:2 IF is an alternation of 2 days of complete or partial fasting a week, and TRE/TRF means eating within a daily period of 12 h or less [25].

Periodic fasting (PF) refers to multiple day, 48–96 h consecutive fasting protocols, such as prolonged water fasting (WF) and fasting mimicking diets (FMD) [22]. FMDs mimic the effects of fasting on markers associated with the stress resistance caused by PF, such as low levels of insulin-like growth factor 1 (IGF-1) and oxidative stress, and consist of fasting and refeeding cycles lasting four or more days in which participants consume a low-protein diet with 30–50% of the normal caloric intake [25].

**Figure 1:**
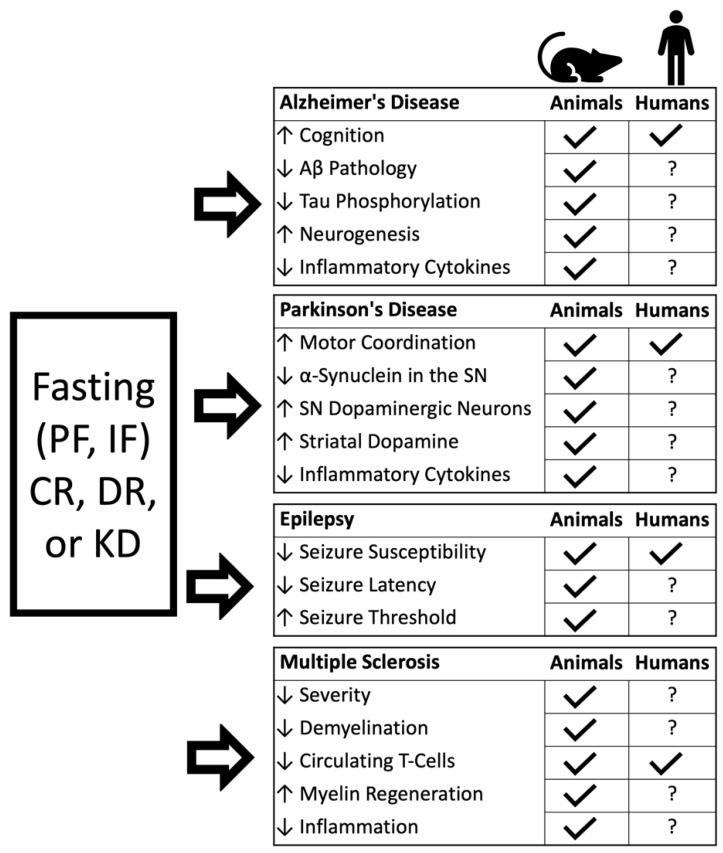
Summary of reviewed findings in animal and human studies of AD, PD, epilepsy, and MS. PF: periodic fasting; IF: intermittent fasting; CR: caloric restriction; DR: dietary restriction; KD: ketogenic diet; Aβ: amyloid beta; SN: substantia nigra.

In this review we will discuss the effects of dietary interventions, with a specific focus on IF and PF, on various aspects of brain aging, as well as their preventive/therapeutic potential in animal studies and clinical trials of AD, PD, epilepsy, and MS (Figure 1). Although dietary interventions have been investigated in animal models of other age-related neurological diseases such as Huntington’s [26,27,28] and Amyotrophic Lateral Sclerosis (ALS) [29,30], only AD, PD, epilepsy, and MS have a comprehensive body of animal studies, as well as some clinical trials. While our review centers on IF and PF as therapeutic interventions, it is necessary to include studies investigating CR and DR, which may have similar mechanisms of action, and which in some cases may be the only dietary interventions attempted in human clinical trials.

## 2. Fasting in Age-Related Neurological Decline

In this section we will review the effects of IF and PF on phenotypic outcomes, such as cognitive function and motor coordination that decline with age, in animal studies and clinical trials. Dietary interventions can impact brain aging-related behavioral outcomes by inducing changes in neurogenesis, synaptic plasticity, neuroinflammation, mitochondrial dysfunction and oxidative stress.

### 2.1. Cognitive Function and Motor-Coordination

Various dietary interventions differing in the age of administration (young, middle age, or old animals), as well as the duration of the intervention have been shown to attenuate cognitive and motor-coordination decline in aging animal models.

In a study by Singh et al. 24 months-old male Wistar rats were either fed ad libitum or an ADF regimen with complete food restriction food every other day for 3 months. At the end of the dietary intervention, rats were tested in the rotarod to assess motor-coordination and the Morris water maze test for spatial memory. ADF rats had a reduced number of falls from the rotarod and increase in time spent on the rotarod, thus indicating better motor-coordination than their age-matched controls. In the Morris water maze, the ADF rats reached the hidden platform in a shorter period of time and spent more time in the target quadrant, both measures of increased spatial memory compared to the control-fed rats [31]. Similarly, 15 months-old Male Wistar rats fed with a 3 month ADF regimen also displayed improvements in motor coordination indicating ADF feasibility even in middle-aged rats [32]. 7–8 week old Sprague Dawley rats were food restricted for 3 weeks by limiting food availability to a 2 h window per day (i.e, TRF). TRF rats ate significantly less calories over time and displayed increased spatial memory in the Barnes maze compared to control-fed rats [33].

11 months of ADF has been shown to improve memory in male CD1 mice when placed on the dietary regimen at 7 weeks of age. In addition, ADF increases the thickness of the CA1 pyramidal cell layer and the expression of dendritic protein (debrin) expression compared to mice on a control diet [34]. Long-term 40% CR for ~18.5 months in female C57BL/6 mice improves spatial memory in the Morris water maze test [35], and in male C57BL/6 mice, 14 to 16 months of 40% CR improves long-term memory assessed by using the Radial Arm Water Maze [36]. 23 months-old female C57Bl/6 mice fed with an FMD (4 days of FMD followed by 10 days of unrestricted refeeding) for 7 months starting at 16 months of age, improve working memory and short-term memory [25]. Using the Barnes maze test of hippocampus dependent long term spatial memory [37], FMD mice employ more of a spatial strategy to find the escape box, make less errors, and have a higher success rate of finding the escape box [25].

In adult rhesus monkeys 30% CR for ~20 years results in a reduction in atrophy in regions of the brain that are important for motor function and aspects of executive function.CR monkeys also significantly maintained lean muscle mass with age compared to monkeys in the control group [38]. 30% CR for 12–17 years also improved executive function [39] and motor performance in aged rhesus monkeys when the CR began at young adulthood [39,40].

In a prospective study of 50 healthy human subjects with a mean age of 60.5 years given either a 30% CR diet (*n* = 19), a diet enhanced with unsaturated fatty acids (*n* = 20) or a control diet (*n* = 10) for 3 months, 30% CR improved verbal memory compared to the other two groups [41]. In a randomized controlled clinical trial of 220 healthy non-obese individuals, a 25% CR diet for two years improved working memory compared to control subjects [42].

Thus, intermittent fasting (ADF and TRF), CR, and FMD improve motor-coordination or cognition in different species from mice to humans.

### 2.2. Synaptic Plasticity and Neurogenesis

Synaptic plasticity refers to modifications in synaptic transmission at pre-existing synapses, while neurogenesis is the creation of new neurons from stem cells that subsequently create new synapses. Both synaptic plasticity and adult neurogenesis are important for continued learning [43] but decline with age [44].

In a study by Talani et al. in Sprague Dawley rats, an increase in spatial memory as a result of TRF, by limiting the availability of food to a 2 h window per day, was accompanied by higher long term potentiation (an electrophysiological measurement of synapse strengthening; [43]) at excitatory synapses at the CA1 region of the hippocampus [33].

The expression of synaptophysin, a marker of synaptic plasticity, increases in the hippocampus of male Wistar rats after 3 months of ADF starting both at 15 months and at 24 months [31,32]. 3 months of ADF starting at 21 months of age in male Wistar rats increases the expression of neural cell adhesion molecule (NCAM) and its polysialylated form (PSA-NCAM) in the hypothalamus, cortex, and the dentate gyrus of the hippocampus [45]. NCAM is associated with learning and memory, as well as regeneration in the adult nervous system [46,47] whereas PSA-NCAM plays a role in the modulation of cell interactions and promotion of synaptic plasticity in the developing nervous system [45].

Brain-Derived Neurotrophic Factor (BDNF) promotes the differentiation of neural progenitor cells into neurons [48,49] and is expressed in the developing and the adult brain [50,51]. BDNF heterozygous knockout (BDNF +/–) mice on an ad libitum diet display reduced neurogenesis compared to mice on ADF and wild type mice on an ad libitum diet, indicating that BDNF is important for mediating the proliferation of neural stem cells. ADF increases the survival of newly generated neural stem cells in wild type mice and to a smaller extent in the BDNF +/– mice indicating that BDNF is a mediator of proliferating stem cell survival by dietary restriction [52].

Brandhorst et al. showed that in the refeeding period following an FMD, the proliferation of immature neurons in the sub granular layer of the dentate gyrus of the hippocampus increases in female C57Bl/6 mice [25].

### 2.3. Neuroinflammation, Mitochondrial Dysfunction, and Oxidative Stress

Chronic low-grade inflammation in tissues is associated with the aging process, including within the brain [53]. Microglia are innate immune cells of the brain responsible for the pathogen defense and for clearing and repairing damage, and their senescence has been implicated in the development of aging-related neurological diseases [54]. Fasting and CR have been shown to decrease inflammation and autoimmunity in many models of aging and disease, and may also delay immunosenescence [25,55,56]. While numerous studies examine the effect of dietary interventions on neuroinflammation in specific disease models, such as AD, few have examined the role of diet in ameliorating neuroinflammation in normal aging. One example is a study by Willette and colleagues in which 19–31-year-old rhesus monkeys having undergone 30% CR for 12–17 years show a reduction in circulating IL-6. Magnetic resonance imaging revealed that IL-6 in the brain is associated with increased brain atrophy [57].

Neurons are the longest living cells in the body and therefore are vulnerable to the accumulation of defective mitochondria [58]. The increase in mitochondrial DNA (mtDNA) mutations over time is one of the driving mechanisms behind mitochondrial aging and the dysfunction of mitochondrial proteins [59]. Multiple studies have shown that fasting and CR induce mitophagy in cells in various tissues, thereby contributing to the removal of dysfunctional mitochondria [60]. The mitochondrial electron transport chain (ETC) is considered to be the main site of endogenous reactive oxidant species (ROS) production, which directly contributes to aging by increasing oxidative stress [61]. ADF in 24-month-old Wistar rats improves cognitive function and ameliorates the age-related functional decline of ETC complex I and IV in mitochondria in the brain [31]. In vivo nuclear magnetic resonance spectroscopy shows that the age-related decline of brain mitochondrial function is reduced in 16-week-old F344BNF1 rats undergoing long term 40% CR [62].

Oxidative stress can impair arterial function during aging through the increase of ROS production (including superoxide anion) and an increase in the enzyme NADPH oxidase that partly mediates this radical. Subsequently, there is a reduced bioavailability of nitric oxide (NO) because of the reaction of superoxide with NO to form peroxynitrite. Peroxynitrite results in an increase in the nitration of tyrosine residues on arterial proteins, mainly by the formation of nitrotyrosine, eventually leading to arterial dysfunction with age [63].

In a study by Donato et al. the effects of lifelong CR typically known to reduce oxidative stress were studied in relation to arterial function. 40% CR in 30-month-old B6D2F1 mice reduces the thickness of the carotid wall compared to control-fed mice and reduces blood pressure. CR also prevents age-related structural changes in the arteries including a reduction in total aortic collagen and an increase in total aortic elastin which declines with age [63]. Along with changes in arterial function, nitrotyrosine and superoxide production is reduced in CR mice, and NO bioavailability is enhanced along with a reduction in superoxide production and NADPH oxidase activity [63].

## 3. The Effect of Dietary Interventions on Neurological Diseases

### 3.1. Alzheimer’s Disease (AD)

Alzheimer’s disease (AD) is the most common form of neurodegenerative disease and the main cause of dementia. AD contributes to 60–70% of all dementia cases in the world. Besides the amyloid beta (Aβ) and tau pathways, current research suggests a strong link between neuroinflammation and AD pathology [64].

Long-term 30% CR prevents the accumulation of Aβ in Tg2576 mice [65], an AD mouse model with mutations in amyloid precursor protein (APP) that results in amyloid plaque development, as well as high levels of amyloidogenic Aβ40 and Aβ42 peptide by 8–10 months of age [66,67]. Schafer et al. also report a reduction of Aβ after 30% CR in Tg2576 mice via a reduced expression of a component of the gamma secretase complex that is responsible for amyloid precursor protein metabolism [68]. In APP mutant mice, ADF improves hippocampal synaptic plasticity through SIRT3 [69].

Along with APP, mutations in the genes encoding integral membrane proteins presenilin 1 (PS1) and presenilin 2 (PS2) are responsible for majority of the cases of familial AD [70,71]. Previous research has shown that PS1 mutations increase the vulnerability of hippocampal neurons to apoptosis [72]. ADF protects hippocampal neurons in male Presenilin (PS1) mutant mice against kainate induced excitotoxic stress [73]. A reduction of Aβ plaques is observed in mice carrying mutations in either APP (J20 mice) or in APP and PS1 (double transgenic mice) after a 40% CR regimen [74]. Mouton et al., report a reduction in Aβ accumulation by observing a decrease in the total volume of Aβ in the cortex and hippocampus following 40% CR for 14 weeks that was given to 13–14 month old mice with mutations in APP and PS1 [75]. Zhang et al. report that ADF for 5 months reduces Aβ deposition and ameliorates cognitive decline in a mouse model carrying mutations in both APP and PS1 starting at 5 months of age [76]. In a study by Wu et al., a 4 month 30% CR regimen improves memory and reduced tau hyperphosphorylation along with resulting in an increase in the expression of genes related to neurogenesis and a reduction in the expression of inflammatory genes in the hippocampus in conditional double knockout PS1 and PS2 mice starting at 4 months of age [77]. CR in a mouse model of Tau deposition (Tg4510 mice) improves short-term memory [78].

40% CR and ADF beginning at 3 months for 14 months in 3xTg mice that exhibit Aβ and tau pathology, improves memory in mice although CR-feeding is more effective in ameliorating Aβ and Tau pathologies compared to ADF [79]. Aβ can impair synaptic function [80] and a previous study by Guo and Mattson showed that a DR mimetic protects rat synapses from Aβ damage [81], thus indicating that long-term ADF in 3xTg mice could act by protecting neurons from synaptic damage even in the presence of Aβ. In another study examining ADF in 3xTg mice, a positive effect was conferred on the neurons by promoting their differentiation and maturation in the hippocampus through GSK-3 Beta. ADF also inhibited insulin and protein kinase A pathways but activated nutrient-sensing AMPK and BDNF pathways [82].

Feeding mice carrying APP mutations starting at 3 months of age with a KD for 43 days reduces Aβ levels along with increasing the levels of ketone bodies. However, it is important to note that the reduction in amyloid levels did not coincide with an attenuation of cognitive decline when the KD is administered for ~1.5 months; indicating that low carbohydrate consumption without substantial CR may not sufficiently improve AD-associated cognitive decline [83]. However, cycles of protein restriction (PRC) improve cognition in 3xTg mice and reduce tau phosphorylation, but do not reduce Aβ levels. Thus, PRC could improve cognitive decline through pathways other than Aβ, such as through a reduction in IGF-1 as well as an increase in insulin-like growth factor binding protein (IGFBP-1) [84]. Contrary to the beneficial effects of CR and ADF described above in multiple mouse models of AD, Lazic et al. observed that ADF administered before the onset of disease has no effect on the degree of plaque deposition, Aβ42 levels, and Blood–brain barrier (BBB) permeability in 5XFAD mice but increases microgliosis and astrogliosis, proinflammatory cytokines, glutamatergic signaling, neuronal injury and reduces synaptic plasticity. The authors state that the mechanism of these negative effects of ADF are unknown, however they suggest that it may be due to the relatively young age of the 2 months-old mice [85].

In a recent study, FMD cycles administered starting at 3.5 months of age in E4FAD mice (5XFAD mice that are homozygous for the apolipoprotein 4 allele) but after the onset of behavioral deficits and when Aβ begins accumulating in the hippocampus and cortex improve spatial memory, increase neurogenesis, reduce Aβ42 levels and amyloid plaques as well as NADPH oxidase (Nox2) levels. Long-term cycles of FMD reduce cognitive decline in 3xTg mice along with a reduction in Aβ load, tau pathology, neuroinflammation, and superoxide generation and an increase in neurogenesis [86].

Obesity has been shown to increase the risk for dementia, however, only few clinical trials have tested the effects of dietary interventions on cognitive impairment. In a single center prospective controlled clinical trial of 80 obese patients with mild cognitive impairment (MCI) either given a CR counseling with nutritionists (*n* = 38) or brief lifestyle counseling (*n* = 37) for 12 months, CR counseling that promoted eating a ~25% CR diet (500 kcal/d) improves cognition while decreasing insulin resistance and inflammation in these patients [87].

Taylor et al. conducted a feasibility study on a short term KD and its effects on cognition in 15 patients with mild AD. 10 out of 15 patients adhered to a KD for 3 months followed by 1 month on a regular diet. Cognition of the AD patients improved during the diet [88].

To test whether FMD cycles can confer similar health benefits as described for mouse models to AD patients, a phase I/II randomized and placebo-controlled (single-blind) clinical study for 40 patients with amnestic mild cognitive impairment (aMCI) or mild AD is ongoing with 28/40 enrolled patients (*n* = 16) in the placebo arm and (*n* = 12) in the FMD arm for a total of 12 FMD cycles (one every two months). Patients in the FMD group are given 5 days of FMD followed by 25 days of normal diet while the patients in the placebo arm are given a diet for 5 days a month in which lunch or dinner was replaced with a meal containing pasta or rice with vegetables and no added supplements. 7/12 patients in the FMD group and 11/16 patients in the placebo group have completed about 6 cycles of the study, indicating that FMD is feasible and tolerable in a small group of AD patients. As the study is ongoing, the enrollment of all AD patients in this clinical trial may provide evidence that FMD cycles can protect against cognitive decline and AD progression in human subjects [86].

For a summary of all AD studies, refer to Table 1.

### 3.2. Parkinson’s Disease (PD)

Parkinson’s disease is the second most common neurodegenerative disorder. The main pathological hallmarks of Parkinson’s disease are intracellular inclusions that contain aggregates of α-synuclein (Lewy bodies), striatal dopamine deficiency, and loss of dopaminergic neurons in the substantia nigra. The motor symptoms of Parkinson’s disease in patients include the presence of bradykinesia, rigidity, and resting tremor. Approximately 90–95% of the cases of Parkinson’s disease are sporadic while the remaining cases are inherited forms of the disease that provide valuable insights into its pathogenesis [89,90,91]. α-synuclein aggregation and mitochondrial dysfunction exacerbate each other and are both important elements in PD pathogenesis [89,90,92]. Peroxisome proliferator-activated receptor-γ co-activator 1α (PGC-1α), a transcriptional regulator of mitochondria, is down regulated in PD [93], and PGC-1α downregulation results in the production of α-synuclein oligomers in vitro [94]. Neuroinflammation is also essential to PD pathogenesis and can promote α-synuclein misfolding [89].

A 3 month ADF regimen starting at 4 months in C57BL/6 mice, before an acute dose of 1-methyl-4-phenyl-1,2,3,6-tetrahydropyridine (MPTP) injections to induce loss of dopaminergic neurons in the substantia nigra (SN) and motor dysfunction, reduces the loss of dopaminergic neurons and improves motor coordination in the rotarod [95]. 30% CR for 27 days in mice injected with MPTP attenuates the loss of dopaminergic neurons in the SN and increases the levels of striatal dopamine in WT but not in ghrelin KO mice, suggesting that the beneficial effect of CR is mediated through ghrelin. In WT mice on the CR regimen, but not ghrelin KO CR mice, higher levels of phosphorylated AMPK and Acetyl-CoA carboxylase (ACC) are observed; thus, suggesting AMPK as a possible target for the neuroprotective effects of ghrelin. Moreover, the selective deletion of β1 and 2 subunits of AMPK specifically in dopaminergic neurons prevents AMPK phosphorylation via ghrelin [96]. In a lactacystin (LAC) mouse model where the loss of dopaminergic neurons and striatal dopamine is due to the disruption of the proteosome, 30% CR is neuroprotective against dopamine neuron degeneration, however, this effect is independent of ghrelin [97].

In another neurotoxin model of PD, ADF for 2 or 8 weeks before 6-hydroxy dopamine (6-OHDA) administration in Sprague-Dawley rats did not prevent the loss of dopaminergic neurons. The authors suggest that the relatively short period of ADF before 6-OHDA lesioning may not be sufficient time to increase neuronal resistance to the neurotoxin [98] or for the stress responses to be activated as fasting usually takes about 2–3 days to activate stress responses [99]. It is also important to note that 6-OHDA causes a 70–80% loss of tyrosine hydroxylase (TH) immune-reactivity (a marker of dopaminergic neurons) which is greater than what is induced by MPTP, and as a result the toxicity of 6-OHDA may have been too strong to be effectively reduced by ADF [100]. Rotenone is another neurotoxin that affects the dopaminergic neurons in the SN. ADF along with rotenone treatment for 28 days in 8-week-old C57BL/6J mice exacerbated dopaminergic neuron degeneration. The mice in the ADF with rotenone group have a decrease in TH^+^ neurons, increase in alpha synuclein accumulation in the SN and an increase in motor dysfunction compared to mice on regular chow treated with rotenone and control mice on regular chow/ADF. The authors propose that this exacerbatory effect of ADF is due to an increase in inflammatory phospholipids such as lysophospholipids and sphingomyelin and excitatory amino acids in the SN [101].

Griffieon et al. studied the effect of ADF versus a high fat diet (HFD) in a genetic mouse model that expresses A53T mutant α-synuclein that can resemble familial PD [102]. These mice exhibit aberrant regulation of the autonomic nervous system (ANS) similar to what is observed in PD patients, characterized by an elevated resting heart rate and the impaired cardiovascular stress response, α-synuclein accumulation in the brainstem, and motor impairment [103]. The ANS abnormality is exacerbated by a high fat diet but improved by ADF [102].

A recent study looked at the effects of 2 FMD cycles before and one cycle after MPTP injections in mice. FMD was found to improve motor-coordination, increase the number of dopaminergic neurons, levels of dopamine, and BDNF. FMD cycles reduce the number of microglia and inflammatory cytokines in the brain. Interestingly, one of the ways through which FMD can bring about these effects is through the modulation of the microbiome [104]. This study confirms the findings of previous research showing that the gut microbiota influences PD pathology and motor deficits in mice that overexpress α-synuclein [105].

Besides rodent models of PD, CR has been administered to rhesus monkeys in order to see if it can exert a neuroprotective effect. 30% CR for 6 months prior to an injection with MPTP increases the survival of dopaminergic neurons in the SN, improves motor activity, and increases the levels of dopamine and glial cell line-derived neurotrophic factor (GDNF) compared to rhesus monkeys on a control diet [106]. GDNF is an important molecule exhibiting neurorestorative as well as neuroprotective properties and can protect dopaminergic cells against toxicity induced by MPTP [107,108].

As different dietary interventions have shown to have potential in ameliorating symptoms of PD, a few clinical trials have been conducted to study its feasibility and efficacy in PD patients.

Vanitallie et al. enrolled 7 PD patients in a feasibility study of the effect of a hyperketogenic diet (HKD). 5 out of 7 patients prepared the HKD at home for 28 days and at the end of the study their Unified Parkinson’s disease score improved [109]. A randomized controlled trial compared a low-fat high carbohydrate diet with a KD for 8 weeks in 47 patients out of which 20/23 completed the low-fat diet and 18/24 completed the ketogenic diet. Both the diets were successful in improving the motor and non-motor symptoms of PD but the KD improved the non-motor symptoms to a greater extent [110].

For a summary of all PD studies, refer to Table 2.

### 3.3. Epilepsy

Epilepsy is a general term for a condition of unprovoked and recurrent seizures with diverse etiologies and therapies. Over 40% of epilepsy cases have no identified cause, and over 30% of patients have conditions that fail to respond to drugs [8]. Fasting was identified as a therapy for epilepsy in ancient times by Hippocrates, but the use of fasting to treat epilepsy did not resurge again until the early 1900s, and has been largely replaced by ketogenic diets starting in the 1920s [111,112]. While KDs are successfully used to treat refractory epilepsy, their mechanisms and effects are different from fasting, CR, and other kinds of DR.

Multiple rodent models of epilepsy have compared CR to KD, finding both similarities and differences. In 10-week-old EL mice, a seizure-prone genetic model, 30% and 15% CR prevents seizures more than an *ad libitum* KD [113]. However, when caloric restriction is adjusted based on body weight, a 38–45% CR standard diet (SD) and 45–52% CR KD in 10-week-old EL mice similarly reduced seizure susceptibility in both diet groups [114].

Using electrophysiological measurements from electrodes implanted in the hippocampus of 5-week-old Sprague-Dawley rats that received 4 weeks of calorie-matched 20% CR, SD or KD, Bough et al. found that both restricted diets induce similar anti-convulsant effects in most methods of stimulation except the duration of maximal dentate activation (MDA) [115]. Hartman et al. attempted to distinguish effects of CR from KD by testing ADF and *ad libitum* KD in multiple acute seizure tests in 3–4-week-old NIH Swiss mice, finding that ADF is detrimental in the 6 Hz and maximal electroshock (MES) seizure models, but protective against kainic acid induced seizures [116]. While Hartman et al. did not find that ADF elevated the seizure threshold of mice injected with pentylenetetrazol (PTZ), Eagles and colleagues reported that 35% CR does elevate the PTZ seizure threshold in 5-week-old Sprague-Dawley rats [117], and another group showed that a 24-h fast reduced the proportion of 6–9-week-old mice developing seizures after a single PTZ dose [118]. Restricting 8-week-old Wistar rats to daily 2-h TRF reduces the proportion of seizures and deaths following pilocarpine injection [119].

One mechanism by which fasting and CR may protect from seizures is by downregulating mTOR in neurons. One month of 15% CR increases AMPK phosphorylation and reduces the phosphorylation of S6, a target of mTOR, in the hippocampus of 3-week-old Wistar rats [120]. Similarly, an increase in AMPK phosphorylation and a decrease in Akt phosphorylation in the hippocampus of rats after 20 days of TRF was observed [119]. 6–9-week old mice with a conditional knockout of DEPDC5 exclusively in neurons (*Depdc5cc+* mice) have constitutive mTOR hyper-activation, increased brain and neuron size, and are more susceptible to PTZ induced seizures and death [121]. Yuskaitis et al. showed that the preventative effect of 24-h fasting on PTZ induced seizures was DEPDC5 dependent, as the seizure incidence of *Depdc5cc+* mice was unchanged by fasting. *In vitro* experiments with neuronal cell cultures evaluating the effects of all amino acids showed that specifically leucine, arginine, and glutamine upregulated mTOR in a DEPDC5 dependent manner, suggesting that an important part of the anticonvulsive effect of 24-h fasting in the brain came from the change in amino acid balance [118].

Many studies have investigated the role of ketones like β-hydroxybutyrate and the reduction of blood glucose in seizure prevention, and the concentrations of the two metabolites are usually inversely correlated. Both Greene et al. and Mantis et al. show that plasma glucose concentrations are correlated with seizure susceptibility in the EL genetic mouse model, and Landgrave-Gomez et al. show that β-hydroxybutyrate is inversely correlated with seizure severity score in a pilocarpine model in rats [113,114,119]. The anti-convulsive effect of elevated β-hydroxybutyrate is considered the underlying mechanism for the use of KD in humans to treat epilepsy, especially when the condition does not respond to drugs [122].

Epilepsy is one of the most common age-related neurological diseases, and the incidence of epilepsy is higher in adults over 60 than in any other age group [123]. Multiple human clinical trials have investigated the effectiveness of KD and less restrictive diets such as the modified Atkins diet (MAD) in preventing epileptic seizures, however nearly all these studies have only involved children [124]. Although KDs and MADs are not the focus of this review, they are included because there was only one small prospective study directly examining IF as a treatment [125], and another which examined the role of PF in initiating KD [126]. Although there have been a few prospective studies, we identified only a single randomized clinical trial to include which examined diet and epilepsy in adults [127]. All other reviewed studies involve children with drug-resistant epilepsy. Bergqvist et al. designed a study to examine the effectiveness of 28-day KD regimens that began with either 24–48 h of fasting or a gradual onset. Initially fasted patients reached ketosis sooner, lost more weight, and experienced more adverse effects, while not significantly altering the overall increase in effectiveness of KD in preventing seizures [126]. In a small (*n* = 6) retrospective study, addition of 5:2 IF for patients already on a KD regimen demonstrated feasibility of IF without clear results [125]. Zare et al. performed the only randomized clinical trial looking at dietary interventions for epilepsy in adults (*n* = 54), and found that 2 months of MAD led to a decrease in seizure frequency 2.19 times that of the control arm [127]. Prompted by the challenges of diet compliance, a large (*n* = 158) clinical trial with children compared the effectiveness of KD, modified Atkins diet (MAD), and low glycemic index therapy (LGIT) diet in preventing seizures in patients. While all dietary interventions showed improvement relative to the control arm, KD was the most effective at reducing seizures [128].

For a summary of all epilepsy studies, refer to Table 3.

### 3.4. Multiple Sclerosis (MS)

Inflammation in MS is characterized by myelin lesions on neurons in the brain and spinal cord which are infiltrated by peripheral immune cells, thereby disrupting axon signaling and producing various neurological symptoms. MS is considered a primarily T-cell mediated disease whose exact pathogenesis is yet unknown, although T-cell autoantigens are currently being discovered [129,130]. Experimental autoimmune encephalomyelitis (EAE) is the most used and validated animal model for MS, where an injection with a known antigen against the myelin sheath, usually myelin oligodendrocyte glycoprotein (MOG) along with an adjuvant induces an autoimmune response [131].

CR and fasting regimens reduce EAE severity and decrease inflammation, demyelination, and axonal damage in multiple studies. Most reviewed studies used CR or ADF for 4–8 weeks before the antigen induction and maintained the diet throughout the disease state [55,132,133,134,135]. Choi and colleagues examined the effectiveness of FMD after EAE induction in 10-week-old C57BL/6 mice, administering three FMD cycles at different start times [136]. In the FMD-fed EAE mice the incidence rate decreases to 46%, compared to 100% for control-fed EAE treated animals, along with a significant reduction in the clinical severity score. 2 cycles of FMD 3 weeks after the first EAE immunization in 6-week-old C57BL/6 mice also presented an effective treatment to ameliorate symptoms [137].

CR, IF, and FMD likely reduce the EAE inflammatory response in part by modulating immune cell populations and inflammatory markers. Both chronic 40% CR [133] and 3 FMD cycles [136] were shown to increase serum corticosterone and reduce some pro-inflammatory cytokines in mice. Both studies by Choi et al. and Bai et al. show that FMD cycles reduce the accumulation of CD4+ T cells and IFN-γ-producing Th1 cells in the spinal cord [136,137]. Following 4 weeks of ADF in 7-week-old C57BL/6 mice, Cignarella et al. found a reduction in IL-17 and IFN-γ producing T-cells in the lymph nodes. The microbiome plays a role in mediating the immune response, as fecal transplants from ADF mice to SD fed mice were able to ameliorate EAE symptoms [135]. Jordan et al. showed that reductions in circulating inflammatory monocytes induced by 4 weeks of ADF in 8-week-old C57BL/6 mice may be mediated by the upregulation of AMPK [55]. Administration of an AMPK activating drug to mice reduced the release of monocytes from the bone marrow into the blood [55].

Fasting and CR appear to mediate immune responses differentially depending on the stimulus. Following 15 days of 66% CR and EAE induction in 6-week-old Lewis rats, Esquifino et al. examined the mitogenic response of proliferating immune cells in the spleen to known activators. While CR decreased the mitogenic response to injection with concavilin A, a T-cell activator, CR did not alter the response to lipopolysaccharide, a bacterial endotoxin [132]. Chronic ADF decreases the expression of pro-inflammatory genes TNFα, IL-1β, CXCL2, and CXCL10 by monocytes in the spinal cord of EAE induced mice [55]. The same study also demonstrated that while ADF reduces the autoimmune response to EAE by decreasing the inflammatory monocyte pool, the immune response to a bacterial infection by *L. monocytogenes* remains unaltered.

Choi et al. and Bai et al. both observe that FMD increases oligodendrocyte regeneration in the spinal cord following autoimmune induced damage. Choi et al. showed that FMD induces regeneration and differentiation of oligodendrocytes. Bai et al. also showed that FMD increases a marker for oligodendrocyte proliferation and reverses EAE induced decreases in the expression of BDNF and other remyelination proteins [136,137].

Recent studies indicate the feasibility and safety of dietary intervention in MS patients, and two studies examining the disease at fundamentally different intervals found changes in immunological parameters in MS patients administered IF [135,138]. Cignarella et al. gave patients 15 days of ADF while they were experiencing an acute MS relapse and observed a decrease in serum leptin and stable or reduced populations of T-cells and B-cells in the blood, consistent with previous animal studies. Fitzgerald et al. administered calorie-matched 5:2 IF and 22% daily CR for 8 weeks in MS patients not experiencing a relapse and found that IF induces significant changes in T-cell subsets whereas CR did not. The observed increase in naïve T-cells and a decrease in memory T-cells in patients following 5:2 IF is consistent with findings by Choi et al. in FMD treated EAE mice [136]. In contrast to Fitzgerald et al. and Choi et al., Cignarella et al. observed that the proportion of naïve T-cells cells decreases, which may have been due in part to the relapse state of the patients and their use of corticosteroid medication [135].

For a summary of all MS studies, refer to Table 4.

## 4. Discussion

Dementia affects 47 million people worldwide, and as the aging population and along with it the prevalence of aging-related diseases continues to grow, that number is anticipated to reach 131 million by 2050 [1]. AD, PD, MS, and epilepsy are chronic diseases that remain without a cure but are a central focus for the development of new treatment modalities, large-scale healthcare incentives, and subsequent funding initiatives. We here summarize that dietary interventions, particularly fasting-based approaches, have the potential to be incorporated as strategies in the prevention and treatments for age-related neurological diseases.

CR and fasting delay aging and aging-related diseases through their modulation of evolutionarily conserved nutrient-sensing pathways. IGF-1 and mTOR are downregulated by fasting, and their inhibition increases autophagy, reduces the accumulation of oxidative damage, and improves insulin sensitivity; all of which are thought to play major roles in the prevention of aging-related diseases [21]. Multiple studies in the disease models we reviewed found changes associated with IGF-1, mTOR, or the related AMPK pathway in AD [82,84], PD [96], MS [55] and epilepsy [118,119,120]. BDNF is a neurotropic factor known to increase neuronal regeneration that has been previously shown to mediate adult neurogenesis by DR in healthy adult mice [52] and BDNF is upregulated after dietary interventions in the brains of animal models of AD [82], PD [104,106], and MS [137]. Therefore, the augmentation of BDNF is of interest as a mechanism by which these interventions may afford protection to people suffering from neurological diseases.

In clinical trials, CR has been shown to improve memory in older individuals [41,42], and cycles of FMD improve many biomarkers of aging [139]. Many of the randomized clinical trials with dietary interventions that we reviewed in this paper showed significant clinical improvements in AD [87,88], PD [110], Epilepsy [126,127,128] and MS [135,138].

Except for Cignarella et al. and Fitzgerald et al. which examined IF regimens in MS, all other reviewed studies with significant clinical improvements were focused on KDs, with some comparisons to LFD [110] and MAD [127,128]. We also reviewed 3 non-randomized smaller prospective studies; including KD trials in AD [87,88] and PD patients [109], and another trial which was the only study to attempt fasting as a potential treatment for epilepsy patients [125]. Another two randomized clinical trials showed the feasibility of FMD without publishing significant clinical improvements. Choi et al. tested one cycle of FMD followed by a Mediterranean diet in MS patients, showing a quality-of-life improvement, while Rangan et al. is still ongoing and awaiting future cognitive and biomarker data on patients with mild AD and aMCI [86,136].

In summary, there is still a significant lack of clinical trials examining the use of fasting interventions for the treatment/amelioration of AD, PD, epilepsy, and MS, despite a growing body of supportive evidence in pre-clinical models. However, there are currently ongoing and unpublished clinical trials in AD [140], PD [141], and MS [142] that are involving therapeutic fasting, and there is promise for more applications of this research in the future.

## Figures and Tables

**Table 1:** All reviewed studies investigating the effects of DR in AD. N/A: relevant data not reported; n/s: relevant data not significant; Aβ: amyloid beta; BHB: beta-hydroxybutyrate; GFAP: glial fibrillary acidic protein; PRCs: protein restriction cycles; IGFBP-1/3: insulin growth factor binding protein- 1/3; IGF-1: insulin growth factor-1; GSK-3β: Glycogen synthase kinase-3 beta; PKA: protein kinase A; NOX2: NADPH oxidase 2; MCI: mild cognitive impairment; aMCI: amnestic mild cognitive impairment.

1 Alzheimer’s Disease Animal Studies						
Model Organism	Age & Sex	Dietary Interventions	Cognitive Function & Neurogenesis	Brain Pathology	Biomarkers	Reference
PS1 mutant knock-in mice	6-weeks male	ADF for 3 months	N/A	↓ neuronal damage	↓ lipid peroxidation product (4-hydroxynonenal)	Zhu et al. 1999 [73]
Tg2576 mice	3 months female	30% CR for 9 months	N/A	↓ Aβ plaques; ↓ Aβ levels	N/A	Wang et al. 2005 [65]
(1) APP (J20) and (2) APP + PS1 mice	(1) 14–15-weeks and (2) 9-weeks male	(1) 40% CR for 2 weeks (2) 40% CR for 15 weeks	N/A	↓ Aβ plaques	n/s	Patel et al. 2005 [74]
APP/V717I mice	3 months female	KD for 43 days	N/A	↓ Aβ levels	↑BHB	Auwera et al. 2005 [83]
3xTgAD mice	3 months male & female	40% CR or ADF for 14 months	↑cognition (40% CR = ADF)	↓ Aβ plaques; ↓ tau phosphorylation (40% CR)	N/A	Halagappa et al. 2007 [79]
PS1 and PS2 double knockout (cDKO) mice	4 months male & female	30% CR for 4 months	↑cognition; ↓brain atrophy	↓ tau phosphorylation	↓ GFAP; ↓ cleaved caspase-3	Wu et al. 2008 [77]
APP and PS1 (dtg APP/PS1)	13–14 months male	40% CR for 14 weeks	N/A	↓ Aβ volume	N/A	Mouton et al. 2009 [75]
3xTg-AD mice	8–9 months male	PRCs for 18–19 weeks	↑ cognition	↓ Tau phosphorylation; ns Aβ plaques	↑ IGFBP-1; ↓ IGF-1; ↓ IGFBP-3; n/s CD11b	Parrella et al. 2013 [84]
Tg4510 mice	18-weeks male & female	CR for 3 months (reduction of body weight to less than 35–40%)	↑ cognition; n/s brain atrophy	N/A	N/A	Brownlow et al. 2014 [78]
Tg2576 mice	2.5 months male & female	30% CR for 2.8 or 12.5 months	N/A	↓ Aβ plaques; ↓ Aβ levels; ↓ gamma secretase complex	N/A	Schafer et al. 2015 [68]
APP/PS1 (B6C3-Tg (APPswe, PS1dE) 85Dbo/J) mice	5 months male & female	ADF for 5 months	↑ cognition	↓ Aβ plaques	N/A	Zhang et al. 2017 [76]
AppNL-G-F knock in mice	12 months male	ADF for either 1, 4 or 12 months.	↑ cognition; ↑ synaptic plasticity	N/A	N/A	Liu et al. 2019 [69]
5XFAD mice	2 months female	ADF for 4 months	↓ synaptic plasticity	↑ neuronal injury; n/s Aβ accumulation	↑IBA-1; ↑GFAP; ↑pro-inflammatory cytokines	Lazic et al. 2020 [85]
3xTg-AD	6 months male	ADF for 3 months	↑ cognition; ↑ neuronal differentiation; ↑ GSK-3β	N/A	↑ AMPK, ↑ BDNF, ↓ PKA, ↓insulin	Li et al. 2020 [82]
(1) E4FAD and (2) 3xTg-AD mice	(1) 3 months female E4FAD (2) 3.5 months (long-term) and 6.5 months (short-term) male and female 3xTgAD	(1) FMD for 4.5 months 2) FMD or 4% PR for 15 months (long-term); FMD for 2 months (short-term)	(1) ↑cognition, ↑neurogenesis (2) ↑cognition, ↑neurogenesis (FMD > 4% PR) (long-term); ↑ cognition (short-term)	(1) ↓ Aβ plaques, ↓ Aβ levels (2) ↓ Aβ plaques, ↓tau hyperphosphoryation (FMD > 4% PR) (long-term); ↓ Aβ plaques, ↓ tau hyperphosphoryation (short-term)	(1) ↓pro-inflammatory cytokines, ↓ NOX2 levels (2) ↓ CD11b (FMD > 4% PR) (long-term); ↓ NOX2 levels, ↓ IBA-1, ↓ pro-inflammatory cytokines (short-term)	Rangan et al. 2022 [86]
**2 Alzheimer** **’s Disease Human Studies**						
**Patient Population**	**Age & Sex**	**Dietary Interventions**		**Clinical Findings**		**Reference**
Obese adults with MCI	60+ (mean: 68) male & female	CR counseling with nutritionists (n = 38) or brief lifestyle counseling (n = 37) for 12 months		↑ global cognition, ↑ memory, ↑ semantic fluency		Horie et al. 2016 [87]
Adults with very mild to moderate AD	(mean: 78) male & female	KD for 3 months followed by 1 month of regular diet		↑ cognition		Taylor et al. 2017 [88]
Adults with aMCI or mild AD	55–80 (mean: 71) male & female	placebo (*n* = 16) and FMD (1 cycle every 2 months) (*n* = 12)		N/A		Rangan et al. 2022 [86]

**Table 2:** All reviewed studies investigating the effects of DR in PD. N/A: relevant data not reported; n/s: relevant data not significant; MPTP: 1-methyl-4-phenyl-1,2,3,6-tetrahydropyridine;.SN: substantia nigra; GDNF glial cell line-derived neurotrophic factor; 6-OHDA: oxidopamine or 6-hydroxydopamine; ANS: autonomic nervous system; p-AMPK: phosphorylated AMP-activated protein kinase; ACC acetyl-CoA carboxylase; IBA-1: ionized calcium-binding adapter molecule 1; GFAP: Glial Fibrillary Acidic Protein; BDNF: brain-derived neurotrophic factor; HKD: hyper ketogenic diet; LFHC low fat high carbohydrate diet.

1 Parkinson’s Disease Animal Studies						
Model Organism (Drug)	Age & Sex	Dietary Interventions	Motor Function & Neurogenesis	Brain Pathology	Biomarkers	Reference
Sprague–Dawley rats (6-OHDA)	male	ADF for 2 or 8 weeks before 6-OHDA injections	N/A	n/s	N/A	Armentero et al. 2008 [100]
C57Bl/6 mice (MPTP)	4 months, male	ADF for 3 months before MPTP injections	↑ motor co-ordination	↑ SN dopaminergic neurons, ↓ SN neuronal damage	N/A	Duan et al. 1999 [95]
THY1-SNCA*A53T mice	12 weeks, male	ADF for 12 weeks	↓ ANS dysfunction, ↑ motor co-ordination	↓ α-synuclein in the brain stem	N/A	Griffioen et al. 2013 [102]
ghrelin WT/KO mice (MPTP)	8 to 10 weeks, male	30% CR for 27 days	n/s	↑ SN dopaminergic neurons (ghrelin WT), ↑ striatal dopamine (ghrelin WT)	↑ p-AMPK, ↑ ACC	Bayliss et al. 2016 [96]
ghrelin receptor WT/KO mice (LAC)	3–4 months and 18–22 months, male	30% CR regimen for 28 days. LAC injections after 21 days on 30% CR	N/A	↑ SN dopaminergic neurons, ↑ striatal dopamine	N/A	Coppens et al. 2017 [97]
C57BL/6J (Rotenone)	8 weeks, male	ADF for 28 days	↓ motor-co-ordination	↓ SN dopaminergic neurons, ↓ striatal dopamine, ↑ α-synuclein in the SN	↓ IGF-1 (IF); ↑ LPC & SM (Rotenone + IF)	Tatulli et al. 2018 [101]
C57Bl/6 mice (MPTP)	7 weeks, male	2 FMD cycles before and 1 FMD cycle after MPTP	↑ motor co-ordination	↑ SN dopaminergic neurons, ↑ striatal dopamine and its metabolites	↓ pro-inflammatory cytokines, ↓ IBA-1, ↓ GFAP, ↑ BDNF	Zhou et al. 2019 [104]
Rhesus monkeys (MPTP)	9–17 years, male	30% CR for 6 months before an MPTP injection	↑ motor co-ordination	↑ SN dopaminergic neurons, ↑ striatal dopamine and its metabolites	↑ GDNF	Maswood et al. 2004 [106]
**2 Parkinson** **’s Disease Human Studies**						
**Patient Population**	**Age & Sex**	**Dietary Interventions**		**Clinical Findings**		**Reference**
Overweight or obese adults with PD (prospective study)	(mean: 61) male & female	HKD (*n* = 5) for 28 days		n/s		Vanitallie et al. 2005 [109]
Adults with PD	(mean: 63) male & female	LFHC (*n* = 20/23) or KD (*n* = 18/24) for 8 weeks		↑ motor and non-motor PD symptoms		Phillips et al. 2018 [110]

**Table 3:** All reviewed studies investigating the effects of DR in Epilepsy. N/A: relevant data not reported; n/s: relevant data not significant; PTZ: pentylenetetrazol; I/O: input/output; MDA: maximal dentate activation; PS: population spike; EEG: electroencephalogram; MES: maximal electroshock.

1 Epilepsy Animal Studies					
Model Organism	Model Organism	Dietary Intervention	Seizure Test	Seizure Findings	Reference
Sprague-Dawley rats	5-weeks male	50% CR, 35% CR, 10% CR, & KD (90%CR) for 20 days	PTZ administered until seizure	↑ seizure threshold (KD = 50% CR)	Eagles et al. 2003 [117]
Sprague-Dawley rats	5-weeks male	15% CR & KD (15% CR) for 28 days	hippocampal electrophysiology: I/O; paired pulse; MDA	↓ PS amplitude (CR&KD); ↑ MDA threshold (CR&KD); MDA latency ↑ CR ↓ KD; ↓ SD events (CR&KD)	Bough et al. 2003 [115]
Wistar rats	3-weeks male	15% CR for 30 days	amygdala electrophysiology: electrical kindling	↑ after-discharge threshold	Phillips-Farfan et al. 2015 [120]
Wistar rats	8-weeks male	TRF (2 h/d) for 20 days	lithium-pilocarpine	↓ seizure score; ↑ seizure latency; ↓ EEG power	Landgrave-Gomez et al. 2016 [119]
EL mice	4 & 10-weeks male & female	30% CR, 15% CR, & KD for 10 weeks	handling induced stress	seizures (↓ 30%CR > ↓ KD)	Greene et al. 2001 [113]
EL mice	10-weeks female	42% CR & KD (48% CR) for 10 weeks	handling induced stress	↓ seizures (CR = KD)	Mantis et al. 2004 [114]
NIH Swiss mice	3–4 weeks male	12 days of ADF & KD (wt. adjusted)	6 Hz test, kainic acid, MES, & PTZ	6 Hz threshold (↓ ADF ↑ KD); kainic acid seizures (↓ ADF); MES threshold (↓ ADF ↑ KD);	Hartman et al. 2010 [116]
Depdc5cc+ mice and littermate control mice	6–9 weeks male & female	24-h fasting	PTZ consistent dose	↑ seizures (Depdc5cc+)	Yuskaitis et al. 2022 [118]
**2 Epilepsy Human Studies**					
**Patient Population**	**Age & Sex**	**Dietary Interventions**		**Clinical Findings**	**Reference**
children with drug-resistant epilepsy	1–14 male & female	24–48 h fast (*n* = 24), or gradual initiation (*n* = 24) before 3 months of KD		↓ seizure frequency (both); ↓ time to ketosis (FAST-KD)	Bergqvist et al. 2005 [126]
children with drug-resistant epilepsy on KD (prospective study)	2–7 male & female	5:2 partial IF (*n* = 6) during KD for 3 weeks-6 months		n/s	Hartman et al. 2013 [125]
adults with drug-resistant epilepsy	age 18–57 male & female	MAD (n = 22) or unchanged diet (*n* = 32) for 2 months		↓ seizure frequency (MAD)	Zare et al. 2017 [127]
children with drug resistant epilepsy	age 1–15 male & female	KD (*n* = 55), MAD (*n* = 58), LGIT (*n* = 57) for 24 weeks		seizure frequency: ↓ 66% KD, ↓ 24% MAD, ↓ 54% LGIT	Sondhi et al. 2020 [128]

**Table 4:** All reviewed studies investigating the effects of DR in MS. N/A: relevant data not reported; n/s: relevant data not significant; EAE: experimental autoimmune encephalomyelitis; SCH: spinal cord homogenate; PLP: proteolipid protein; MOG: myelin oligodendrocyte glycoprotein; CNS: central nervous system; BHB: beta-hydroxybutyrate; RRMS: relapsing remitting multiple sclerosis; QOL: quality of life.

1 Multiple Sclerosis Animal Studies					
Model Organism (Antigen)	Age & Sex	Dietary Interventions	Clinical Findings	Immune & Biomarkers	Reference
Lewis rats, EAE (SCH)	6-weeks male	66% CR for 15 days pre-induction	↓ clinical score	↓ spleen & lymph IFN-γ	Esquifino et al. 2007 [132]
SJL mice, EAE (PLP)	5-weeks female	40% CR for 5 weeks pre-induction	↓ clinical score; ↑ survival; ↓ spinal cord inflammation, demyelination, & axon damage	↑ corticosterone & adiponectin; ↓ leptin & IL-6	Piccio et al. 2008 [133]
C57BL/6 mice, EAE (MOG)	13-weeks female	ADF for 8 weeks pre-induction, post-induction, or both	↓ incidence (ADF post)	N/A	Kafami et al. 2010 [134]
C57BL/6 mice, EAE (MOG)	10-weeks female	FMD (3 cycles) at 10% symptomatic (S), at 100% symptomatic (T), or at 2 weeks post-symptoms (C-F)	↓ clinical score (all); ↓ incidence (S); ↓ spinal cord inflammation & demyelination (T); ↑ myelin regeneration (T)	↓ blood WBC, lymphocytes, monocytes, & granulocytes; ↓ CNS CD4 & CD8 T-cells; ↑ spleen naïve:active CD4 T-cell ratio (T)	Choi et al. 2016 [136]
C57BL/6 mice, EAE (MOG)	7-weeks female	ADF for 4 weeks pre-induction	↓ clinical score; ↓ spinal cord inflammation & demyelination;	↓ lymph T-cell IL-17 & IFN-γ; ↑ corticosterone & BHB; ↑ T-regs in small intestine	Cignarella et al. 2018 [135]
C57BL/6 mice, EAE (MOG)	8 weeks-female	ADF for 4 weeks pre-induction	↓ clinical score	↓ blood & CNS inflammatory monocytes; ↓ CNS monocyte TNFα, IL-1β, CXCL2 &CXCL10	Jordan et al. 2019 [55]
C57BL/6 mice, EAE (MOG)	6-weeks female	FMD (2 cycles) at 3 weeks post-induction	↓ incidence; ↓ clinical score; ↓ spinal cord inflammation & demyelination; ↑ myelin regeneration	↓ spinal cord leukocytes & macrophages; ↓ CNS CD4 & CD8 T-cells; ↓ CNS T-cell IL-17 &↑ IFN-γ; ↑ spleen CD4 & CD11b T-cells	Bai et al. 2021 [137]
**2 Multiple Sclerosis Human Studies**					
**Patient Population**	**Age & Sex**	**Dietary Interventions**	**Clinical Findings**	**Immune & Biomarkers**	**Reference**
Adult RRMS patients	18–68 (mean: 45) male & female	1-cycle of FMD followed by a Mediterranean diet (*n* = 18), KD (*n* = 18), or control diet (*n* = 12) for 6 months	↑ QOL (FMD & KD)	n/s	Choi et al. 2016 [136]
Adult RRMS patients undergoing relapse	18–60 (mean: 41) male & female	<500 kcal ADF (*n* = 8) or control diet (*n* = 8) for 15 days	n/s	↓ blood T&B cells; ↓ naïve CD4 T-cells; ↓ Leptin	Cignarella et al. 2018 [135]
Adult RRMS patients not undergoing relapse	18–50 (mean: 37) male & female	75% CR 5:2 IF (*n* = 11), 22% CR (*n* = 11), or control diet (*n* = 9) for 8 weeks	n/s	↓ memory T-cells and Th1 cells ↑ naïve CD4 T-cells	Fitzgerald et al. 2022 [138]
